# Retrospective assessment of barriers and access to genetic services for hereditary cancer syndromes in an integrated health care delivery system

**DOI:** 10.1186/s13053-022-00213-5

**Published:** 2022-02-10

**Authors:** Kristin R. Muessig, Jamilyn M. Zepp, Erin Keast, Elizabeth E. Shuster, Ana A. Reyes, Briana Arnold, Chalinya Ingphakorn, Marian J. Gilmore, Tia L. Kauffman, Jessica Ezzell Hunter, Sarah Knerr, Heather S. Feigelson, Katrina A. B. Goddard

**Affiliations:** 1 Department of Translational and Applied Genomics, Center for Health Research, Kaiser Permanente Northwest, 3800 N Interstate Avenue, Portland, OR 97227 USA; 2 Center for Health Research, Kaiser Permanente Northwest, 3800 N Interstate Avenue, Portland, OR 97227 USA; 3 Department of Health Services, University of Washington, 1959 NE Pacific Street, Seattle, WA 98195 USA; 4 Institute for Health Research Kaiser Permanente Colorado, 10065 E Harvard Avenue #300, Denver, CO 80231 USA

**Keywords:** Genetic evaluation, Genetic referrals, Hereditary breast and ovarian cancer, Lynch syndrome, Genetic testing

## Abstract

**Background:**

A critical step in access to genetic testing for hereditary cancer syndromes is referral for genetic counseling to assess personal and family risk. Individuals meeting testing guidelines have the greatest need to be evaluated. However, referrals to genetics are underutilized in US patients with hereditary cancer syndromes, especially within traditionally underserved populations, including racial and ethnic minorities, low-income, and non-English speaking patients.

**Methods:**

We studied existing processes for referral to genetic evaluation and testing for hereditary cancer risk to identify areas of potential improvement in delivering these services, especially for traditionally underserved patients.

We conducted a retrospective review of 820 referrals to the Kaiser Permanente Northwest (KPNW) genetics department containing diagnosis codes for hereditary cancer risk. We classified referrals as high- or low-quality based on whether sufficient information was provided to determine if patients met national practice guidelines for testing. Through chart abstraction, we also assessed consistency with practice guidelines, whether the referral resulted in a visit to the genetics department for evaluation, and clinical characteristics of patients receiving genetic testing.

**Results:**

Most referrals (*n* = 514, 63%) contained sufficient information to assess the appropriateness of referral; of those, 92% met practice guidelines for genetic testing. Half of referred patients (50%) were not offered genetic evaluation; only 31% received genetic testing. We identified several barriers to receiving genetic evaluation and testing, the biggest barrier being completion of a family history form sent to patients following the referral. Those with a referral consistent with testing guidelines, were more likely to receive genetic testing than those without (39% vs. 29%, respectively; *p* = 0.0058). Traditionally underserved patients were underrepresented in those receiving genetic evaluation and testing relative to the overall adult KPNW population.

**Conclusions:**

Process improvements are needed to increase access to genetic services to diagnose hereditary cancer syndromes prior to development of cancer.

## Background

A critical step in access to genetic testing for hereditary cancer syndromes is a referral for genetic evaluation to assess personal and family risk. Referral to genetics is underutilized in US patients, particularly within underserved populations [1–3]. Indeed, it is estimated that less than 1.2% of individuals with Lynch syndrome (LS) and only 6% of individuals with pathogenic variants in *BRCA1/2* have been identified in the general population [4–6]. These figures are troubling given that practice guidelines developed by leading cancer, genetics, and prevention-focused organizations highlight the importance of identifying individuals with hereditary cancer syndromes [7, 8]. The consequences of failing to identify at-risk individuals include missed opportunities to offer risk-reducing strategies [9, 10], which are especially prevalent in traditionally underserved populations [11, 12].

Understanding of how patients are referred to genetic services (evaluation and receipt of genetic testing, as appropriate, by a genetics provider) and whether guidelines for genetic testing are followed is necessary to ensure efficient and effective delivery of care. While there is increasing demand for genetic services related to testing for hereditary cancer syndromes [13, 14], interest is not limited to high-risk patients, and is outpacing the capacity of genetics practitioners [15, 16]. Increase in referral volume can lead to long delays for appointments for these patients. An efficient process that prioritizes guideline-appropriate referrals could help ensure equitable provision of this valuable resource.

We conducted a retrospective review of referrals to the Kaiser Permanente Northwest (KPNW) genetics department during a seven-month period in 2017. This research was part of the Cancer Health Assessments Reaching Many (CHARM) study. CHARM is one of six clinical sites of the Clinical Sequencing Evidence-Generating Research (CSER) consortium (https://cser-consortium.org/), a national multi-site research program funded by the National Human Genome Research Institute (NHGRI) with co-funding from the National Institute on Minority Health and Health Disparities (NIMHD) and the National Cancer Institute (NCI) [17]. CHARM examined a multi-faceted intervention to increase access to genetic testing for hereditary cancer syndromes in populations that have been traditionally underserved by medical advances. Understanding the baseline performance of the traditional genetics service model is critical to identifying and assessing the effectiveness of interventions. In this study, we aimed to evaluate referral quality to document the potential consequences of limiting access to genetics evaluation on the basis of referral quality. Here we examined referrals to the KPNW genetics department prior to the implementation of the CHARM intervention to identify areas for improvement in the delivery of genetic evaluation and testing for hereditary cancer risk, especially among traditionally underserved patient populations.

## Methods

### Setting

KPNW is an integrated health care delivery system serving approximately 625,000 members in the greater Portland, Oregon area. The study was approved by the Institutional Review Board at KPNW*. *No informed consent was requiredfrom subjects as data were anonymously extracted from the EMR. In accordancewith the Oregon Genetic Privacy law, individuals were either offered theopportunity to opt-out of anonymous research or were excluded from analyses.All procedures were in accordance with US Federal Policy for the Protection of Human Subjects.

### Study subjects

Patients can be referred to the genetics department through their primary care provider, a specialty care provider, or through self-referral. While multiple syndromes can lead to hereditary cancer, we limited our review to adult referrals where the indication included a personal and/or family history of cancers associated with the most common hereditary cancer syndromes, Hereditary Breast and Ovarian Cancer syndrome (HBOC) and Lynch syndrome (LS) because of the greater familiarity of non-genetics specialists with these conditions and their associated referral criteria. Study genetic counselors (JZ, MG) reviewed a list of all diagnosis codes associated with referrals and selected cases for inclusion. We included referrals if they contained diagnosis codes for invasive and in situ female and male breast cancer, ovarian cancer (including fallopian tube cancer), endometrial cancer (of any subtype), colorectal cancer, colon polyps, stomach cancer, pancreatic cancer, and renal cancer. We excluded referrals for a family or personal history of thymoma, “lymph node cancers”, lung cancer, pheochromocytoma, and renal cell carcinoma, in order to limit our review to cancers related to the subset of guidelines that were part of our analysis.

Evaluated referrals were issued from June through December of 2017. We obtained demographic data from Electronic Medical Records (EMR). We defined patients as underserved if they were residents of a medically underserved area, if they were listed as racial and ethnic minority (Hispanic or race other than White) in the EMR, if they were on Medicaid, or if they had a primary language other than English listed in the EMR. Patient history of cancer prior to the referral date was assessed using tumor registry records.

### Genetic clinical practice

At the time of the study genetics department practice was to send a detailed family history form to the patient following receipt of the referral. Patients were then invited for an initial genetic counseling visit after returning the family history form; and if they had an urgent clinical need for assessment their appointment may be scheduled before completion of the form. At the initial genetic counseling visit, a genetics provider (genetic counselor or geneticist) evaluated family history to verify the information provided by the patient in the form and elicit further details, and offered genetic testing if deemed clinically appropriate. Genetic testing results were returned by the genetics provider in person or over the phone and documented in the EMR.

### Referral assessment

We categorized referrals by quality and by consistency with guidelines.

*Assessing Quality:* Quality of an eligible referral was classified as high or low by the genetic counselors (JZ, MG) based on the level of detail provided in the referral related to the patient’s personal and family cancer history through diagnosis codes. Referral quality was determined by assessing the provided documentation of patient’s: 1) age at diagnosis; 2) sex; 3) primary cancer site (if applicable); 4) relationship to an index case in the family (if other relatives previously tested positive); and 5) number and age of affected and unaffected relatives. A high-quality referral had to include clinical characteristics to determine whether the patient met national practice guidelines for hereditary cancer genetic evaluation and testing [7, 8]. Referrals not providing enough information to decide if genetic evaluation and testing should be offered, were classified as low-quality referrals. The genetic counselors (JZ, MG) divided the task of reviewing the referrals and resolved questions by discussion.

*Assessing Consistency:* One genetic counselor (JZ) assessed each high-quality referrals consistency with clinical guidelines at two time points: at the time of referral, and following initial evaluation of the patient by a genetics provider. Assessment following the initial evaluation was based on EMR documentation abstracted via chart review. Consistent referrals were high-quality referrals where the patient met at least one criterion for referral for cancers associated with HBOC or LS based on the 2015 professional guidelines from NSGC/ACMG [18]. A non-consistent referral was a high-quality referral that did not meet any criteria for referral. Low-quality referrals were coded as unknown.

### Manual Chart Review

 We conducted detailed chart abstraction of all eligible referrals using a standardized form implemented in REDCap [19]. Chart abstraction identified: 1) clinical referral process steps; 2) if the patient returned a family history form; 3) if a genetic counseling visit for evaluation was scheduled; 4) if the patient attended a genetic counseling visit; 5) if genetic testing was offered; 6) if genetic testing was completed; 7) genetic test type and results (unknown results were coded as negative); and 8) whether the patient received the test results.

### Statistical analysis

Demographic and clinical characteristics of referred patients were compared to the overall population of KPNW adult members in early 2020 using Chi-Square tests (for categorical variables) and t-tests (for continuous variables). We assessed the flow of patients through the referral and testing process with descriptive statistics. Differences in referral quality by provider specialty were assessed using logistic regression. Differences in referral outcomes were assessed using Chi-Square tests. We used a significance threshold of *p* < 0.05 for all analyses. Statistical analysis was conducted using SAS 9.4 (SAS Institute Inc., Cary, NC).

## Results

**Fig. 1:**
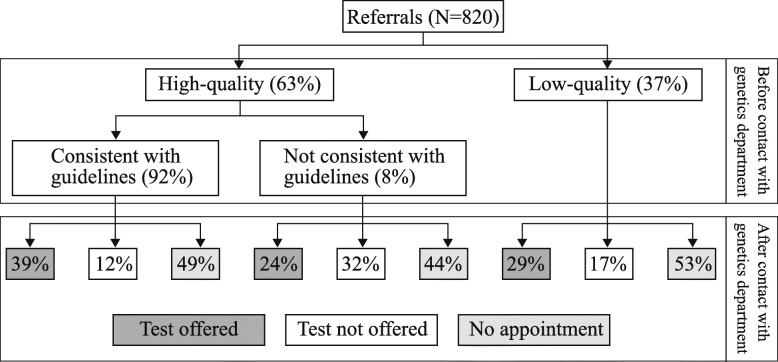
Referral quality and compliance to Kaiser Permanente Northwest genetics department for hereditary cancer risk evaluation. A referral was defined as high-quality if it included enough information to determine if the patient should be offered genetic services based on clinical guidelines. Prior to contact with the genetics department is defined as using personal and family cancer history included in the diagnosis codes and referral notes. After contact with genetics department is defined as using information obtained during a pre-test counseling visit

A total of 820 eligible patients were referred to the genetics department during the study period. The referred population was more likely to be White, non-Hispanic and female, and to speak English as their primary language, compared to the overall KPNW population (Table 1). About 30% of referred patients had a personal history of cancer. Referred patients had a mean of 14.9 years of KPNW membership. Most referrals to the KPNW genetics department were high-quality (63%), and 92% of high-quality referrals were consistent with clinical practice guidelines (Fig. 1). Forty-one percent of referred patients did not complete the family history form, and as a result, about half of all referred patients (*n* = 368, 45%) were not offered an appointment with a genetics provider. However, 143 patients with low-quality referrals had an appointment scheduled. Of these, 53 were not offered genetic testing. Patients with high-quality, consistent referrals were more likely to evaluated and offered genetic testing than patients with low-quality referrals (39% vs. 24%, respectively; *p* = 0.0058).

**Table 1:** Characteristics of patients referred to genetic services compared to overall adult Kaiser Permanente Northwest membership.

	Referral cohort Age 18+ *N* = 820		KPNW current members Age 18+ *N* = 500,924		
	N	%	N	%	*p*-value*
**Sex**					<.0001
Female	761	93	262,068	52	
Male	59	7	238,370	48	
**Age**					<.0001
18–49	416	51	270,933	54	
50–64	262	32	123,918	25	
65+	142	17	106,073	21	
**Underserved**	219	27	153,940	31	0.0126
Racial/Ethnic minority**	107	13	93,121	19	<.0001
Primary language other than English	18	2	25,474	5	0.0002
Medicaid coverage	47	6	32,689	11	0.36
Resident of medically underserved area	82	10	45,843	9	0.40
**Years of KPNW membership**					
Mean / Std	14.9 yrs	12.5 yrs	12.7 yrs	12.0 yrs	<.0001
**Personal history of cancer**					
Any of types listed below	252	31	13,250	3	<.0001
*Specific type, not mutually exclusive*					
Colorectal	15	2	1658	0	<.0001
Endometrial	31	4	1100	0	<.0001
Other Lynch syndrome related***	9	1	4346	1	0.48
Breast, male	3	0	24	0	<.0001
Breast, female	184	22	6206	1	<.0001
Ovarian	27	3	240	0	<.0001

* p-value from Chi square test, except ‘Years of KPNW’ membership, which comes from t-test

** Hispanic or race other than White

*** Other Lynch syndrome related: stomach, small intestines, intrahepatic bile ducts, renal pelvis, sweat gland/sebaceous carcinoma

**Fig. 2:**
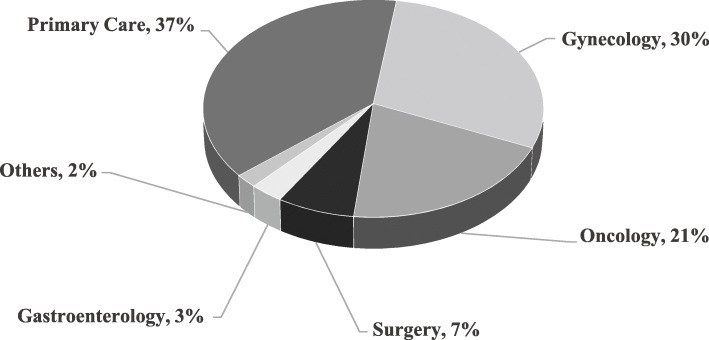
Referrals to Kaiser Permanente Northwest genetics department by provider department. Others include self-referrals and referrals from other departments including dermatology, endocrinology, urgent care, urology, palliative care, and pediatrics

**Fig. 3:**
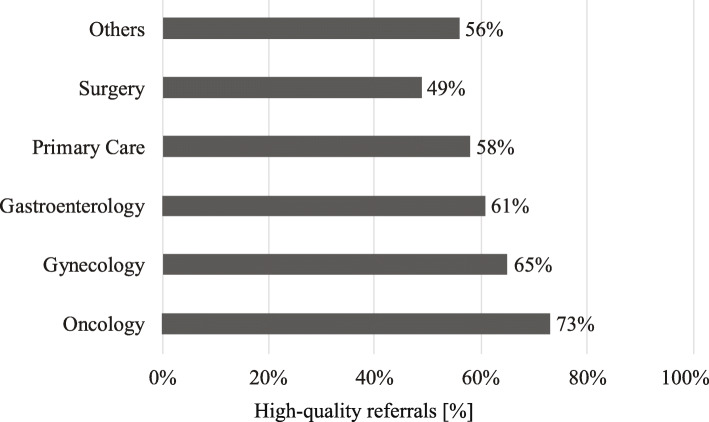
Proportion of high-quality referrals by referring department at Kaiser Permanente Northwest. Others include self-referrals and referrals from other departments including dermatology, endocrinology, urgent care, urology, palliative care, pediatrics

Primary care (37%), gynecology (30%), and oncology (21%) providers made the most genetics referrals (Fig. 2). A higher percentage of referrals made by oncology were high-quality than referrals from primary care (73% vs. 58%; *p* = 0.0016) (Fig. 3). Patients referred from oncology and surgery were more likely to have a personal history of cancer (92 and 52%, respectively), than the patients referred from other departments.

**Fig. 4:**
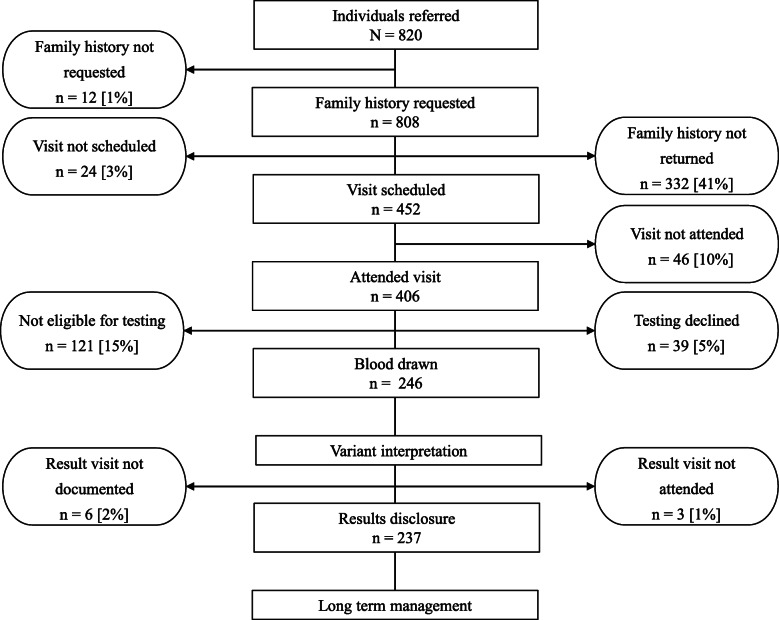
Outcome of patient referrals. Most patients do not complete all the necessary steps to receive genetic services, especially early in the process by not returning the family history form. Each step shows where patients dropped off the process and were lost to further follow-up. Rounded percentages are relative to the individuals referred. Clinic and provider factors are shown on the left and patient factors are shown on the right side of the figure

Overall, more than two-thirds of patients referred to the genetics department did not get genetic testing (Fig. 4). Underserved patients (*n* = 219) were equally as likely to be referred as the overall population and we did not observe differences in the outcome of referrals between these two groups. The largest share of patients who did not attend a genetic counseling visit to be evaluated (41% overall and 43% underserved) were lost to further follow-up because they did not return a family history form. Of the 406 individuals attending a visit, 70% (69% of the 105 underserved individuals) were offered genetic testing; this made up 35% of all referrals and 33% of referrals for underserved patients. Among those who received testing (overall *n* = 246, underserved *n* = 64), 66% of those from underserved groups had a personal history of cancer, compared to 57% of those who were not known to be from an underserved group (*p* = 0.21).

There were two primary reasons that people were not offered testing: 1) they did not meet guidelines based on additional family history information (74% in high-quality and 92% in the low-quality group); or 2) it was documented by the genetics provider that another more informative family member should be tested first (19 and 8%, of those not offered testing in the high- and low-quality groups, respectively); these rates were similar in the overall population and in the underserved population.

Most tested patients were offered a cancer gene panel (89%); the remaining 11% were offered a test for a known familial variant. Thirty-nine patients were offered genetic testing but did not complete the test (14 active refusals, 24 passive refusals (did not complete blood draw); one patient died prior to testing).

Overall, 18% of genetic test results were positive, identifying pathogenic or likely pathogenic variants; 25% identified variants of unknown significance (VUS); and the remainder were negative. The rate of positive results was not statistically different by referral quality (high-quality: 19%; low-quality: 15%; *p* = 0.49).

## Discussion

Our study identified several barriers to accessing genetic evaluation and testing for hereditary cancer syndromes in an integrated healthcare system setting. First, requiring completion of a family history form before scheduling an appointment appeared to be a major access barrier. Forty-one percent of referred individuals (including 43% of those categorized as underserved) did not return the form, accordingly, most of them were not offered genetic evaluation. Second, one in five patients who returned the family history form did not complete all the steps required to access genetic services, from not attending a scheduled evaluation visit through receiving their test results. These are missed opportunities, especially since 55% of these patients appear to meet criteria for testing. Third, about one-third of referrals did not include enough information to determine if the patient should be offered genetic counseling to be evaluated based on clinical guidelines, but the outcome of testing did not differ by referral quality. Fourth, patients who received referrals to the genetics department were not representative of the overall patient population: those receiving referrals were more likely to be non-Hispanic and White and female than the overall KPNW population, suggesting under-referral of underserved populations.

Our analysis shows that requiring patients to complete and return a family history form creates a bureaucratic burden resulting in a major access barrier, even for individuals with high-quality and guideline-consistent referrals. Many patients for whom testing would be clinically appropriate did not return the form and thus were not scheduled for a genetics evaluation. Further, the information on this form was not used to prioritize or limit appointments among patients who completed and returned the form.

One solution to the barriers to testing identified here would be to simplify referral criteria. Universal screening programs that systematically screen all individuals with a new diagnosis ensure greater equity in access to genetic evaluation and testing than programs that require additional criteria be met [20–23]. This may be particularly important considering the high positive genetic testing rate we observed of 18%, which far exceeds the population prevalence of these conditions, and the lack of statistical difference between outcomes of high- and low-quality referrals. Guidelines that are complex, with multiple criteria that are difficult to remember, hinder the use and systematic implementation of genetics referrals and testing in clinical practice, and many individuals with genetic risk may be missed using the traditional familial and risk-based approach [24]. Additional options for improving access include using an online family history questionnaire (provider or patient facing) or a chatbot as a more structured data collection tools [25–27], or providing paper-based tools with simplified rules for referral and systematic scoring, as endorsed by the United State Preventative Service Task Force [28]. Further, broader testing approaches by health care systems as well as a streamlined pre-test counseling process may allow for reduced wait times and increased identification of patients eligible for testing and expanded general access to care [29].

Even after receiving a genetics referral, patients face numerous challenges such as low awareness and knowledge of genetic testing, accessibility of genetic service, or mistrust among others, as they attempt to follow through with the referral [30]. Once genetic testing was offered, 86% of patients overall and 89% of underserved patients had their sample collected for genetic testing – rates of testing did not differ significantly between underserved and majority groups. Others have also reported similar rates of genetic testing among underserved and more advantaged groups once patients are referred and evaluated [31–33]. Patient perceptions about the referral process influence their decision to seek evaluation and testing. Patients may decline subsequent steps if they do not feel at increased genetic risk, if another family member has tested negative, or if the referring provider is non-directive or dismissive of the referral [34].

Our analyses of referral quality indicate that low-quality referrals were not indicative of patients not qualifying for genetic testing. We observed that the rate of positive genetic test results did not differ between high- and low-quality referrals. It may be easier for a provider such as an oncologist to make a high-quality referral when a patient meets referral guidelines based on their personal history of cancer compared to a referral is based solely on family history. Our results support the idea that the health system should expand genetics capacity or use strategies to ensure genetic evaluation and testing for all who might benefit that do not place undue burden on providers (such as by limiting access by referral quality) or on patients (such as by requiring lengthy family history forms).

The highly significant difference (*p* < .0001) between the proportion of referred members who were documented as racial/ethnic minorities in the EMR (13%) and the proportion in the overall KPNW adult population (19%) strongly suggests disparities in access to referrals between racial and ethnic groups [35]. While our data do not allow us to directly compare the racial/ethnic makeup of those referred to genetics to KPNW members who were eligible but not referred, our findings are consistent with past studies that have documented that women from traditionally underserved groups, including African American and Hispanic women, are less likely to receive genetic referral and testing than White, non-Hispanic women [31, 36, 37]. Even as utilization of breast and colorectal cancer screening increases, racial disparities persist [38]. Our study, similar to what was reported by Peterson et al., found that disparities start at the initial referral stage [11]. These findings highlight that traditionally underserved patients already have less contact with their providers than more advantaged patient groups even prior to the referral stage [39, 40]. It is critically important to increase healthcare access to ensure equitable use of genetic testing among all patient populations and to address additional factors including differences in knowledge of family history of cancer [41, 42], awareness of genetic evaluation and testing, uncertainty about the information provided, and medical mistrust [3].

Our study echoes the results of several prior studies in showing that patients with a known personal and family history of cancer are more likely than those without to be referred to genetics departments, regardless of their race or ethnicity [6, 43]. Thus, Yedjou et al. [44] reports that patients from underserved populations are more likely to be referred and receive genetic testing having advanced-stage cancer, while patients who are non-Hispanic White are more likely to be referred due to known family cancer history as described by Chapman et al. [9].

The under-identification of people with hereditary cancer syndromes prevents individuals from receiving potentially life-saving interventions to reduce or mitigate their cancer risk [41]. While it may not be surprising that female patients are more likely to seek or receive a referral to genetics given that many patients were referred due to potential risk for HBOC, this nonetheless represents a care gap for men, who can also be affected by these hereditary cancer syndromes. An average KPNW membership of 14.9 years at the time of referral in this study demonstrates that not only is there ample opportunity to identify individuals with hereditary cancer syndromes sooner, perhaps before developing cancer, but that these individuals are likely to remain members in the health system and quite possibly need cancer treatment in the future if preventive measures are not taken.

### Limitations and future directions

This study has some important limitations. First, we only included a single integrated health care system during seven months in 2017. Additionally, we were unable to determine consistency with guidelines for low-quality referrals when additional genetic counseling was not offered. We also may have underestimated the proportion of low-quality referrals by excluding referrals that did not contain personal or family history diagnosis codes for cancers associated with hereditary risk from our analyses. Finally, our study population had limited racial and ethnic diversity. Despite these limitations, this study provides important data on genetic counseling referrals that will be essential to decreasing the burden of hereditary cancer syndromes. Our study was conducted in an integrated health system with standardized policies for coverage of hereditary cancer genetic tests and genetic counseling, and an existing genetics department, eliminating many of the barriers faced in other systems.

New approaches are needed to improve the efficiency and effectiveness of identifying individuals with hereditary cancer syndromes, especially those from traditionally underserved populations. Solving initial referral barriers is not sufficient to address all challenges that patients face. Additional interventions are needed to ensure that patients can complete all the steps in the process of seeking out and accessing genetic evaluation and testing, allowing them to reap the benefits of learning hereditary cancer risk information.

Future studies should evaluate automated tools to assess risk and provide patient and provider education aiming to increase referral quality [42, 45, 46] as well as to assess if streamlining the pre-test genetic counseling process might result in improved and timely access to genetic testing. Online consent, telehealth visits, and pre-education may allow more patients to follow through with receiving genetic referral and reduce burden for healthcare providers. Additional research should test how different referral strategies impact identification of individuals with hereditary cancer syndromes to ensure that they do not have unintended consequences that hinder access. Findings of studies like CHARM, which will examine the use of an online risk assessment tool, might also offer opportunities to increase access to genetic testing for hereditary cancer syndromes, especially in populations that have been traditionally underserved by the medical system.

## Conclusions

Process improvements are needed to ensure equitable access to genetic evaluation and to increase identification of individuals with hereditary cancer syndromes. Overcoming initial obstacles, such as insurance coverage or referral, is not sufficient. Strategies are needed to streamline and support patients in completing all steps required to access genetic evaluation and testing to maximize the benefits of learning personal genetic risk information. Ideally, genetics providers should model their service delivery to handle larger numbers of patients, minimizing the need to pre-screen patients through family history forms or by referral quality.

## Data Availability

The datasets generated and/or analyzed during the current study are not publicly available due to privacy and ethical restrictions but are available from the corresponding author on reasonable request.

## Abbreviations

KPNW: Kaiser Permanente Northwest
HBOC: Hereditary breast and ovarian cancer syndrome
LS: Lynch syndrome
EMR: Electronic Medical Records
CHARM: Cancer Health Assessments Reaching Many study
CSER: Clinical Sequencing Evidence-Generating Research consortium
NSGC: National Society of Genetic Counselors
ACMG: American College of Medical Genetics and Genomics

## References

[1] Evans O, Gaba F, Manchanda R (2020). Population-based genetic testing for Women's cancer prevention. Best Pract Res Clin Obstet Gynaecol.

[2] Sutton AL, Hurtado-de-Mendoza A, Quillin J, Rubinsak L, Temkin SM, Gal T, Sheppard VB (2020). Reducing disparities in receipt of genetic counseling for underserved women at risk of hereditary breast and ovarian cancer. J Women's Health (Larchmt).

[3] Williams CD, Bullard AJ, O'Leary M, Thomas R, Redding TS, Goldstein K (2019). Racial/ethnic disparities in BRCA counseling and testing: a narrative review. J Racial Ethn Health Disparities.

[4] Bellcross C, Hermstad A, Tallo C, Stanislaw C (2019). Validation of version 3.0 of the breast Cancer genetics referral screening tool (B-RST). Genet Med.

[5] Couch FJ, Shimelis H, Hu C, Hart SN, Polley EC, Na J, Hallberg E, Moore R, Thomas A, Lilyquist J, Feng B, McFarland R, Pesaran T, Huether R, LaDuca H, Chao EC, Goldgar DE, Dolinsky JS (2017). Associations between cancer predisposition testing panel genes and breast cancer. JAMA Oncol.

[6] Wood ME, Kadlubek P, Pham TH, Wollins DS, Lu KH, Weitzel JN, Neuss MN, Hughes KS (2014). Quality of cancer family history and referral for genetic counseling and testing among oncology practices: a pilot test of quality measures as part of the American Society of Clinical Oncology quality oncology practice initiative. J Clin Oncol.

[7] Owens DK, Davidson KW, Krist AH, Barry MJ, Cabana M, U. S. Preventive Services Task Force (2019). Risk assessment, genetic counseling, and genetic testing for BRCA-related cancer: US Preventive Services Task Force recommendation statement. JAMA..

[8] Gupta S, Provenzale D, Llor X, Halverson AL, Grady W, Chung DC, Haraldsdottir S, Markowitz AJ, Slavin Jr TP, Hampel H, Ness RM, Weiss JM, Ahnen DJ, Chen LM, Cooper G, Early DS, Giardiello FM, Hall MJ, Hamilton SR, Kanth P, Klapman JB, Lazenby AJ, Lynch PM, Mayer RJ, Mikkelson J, Peter S, Regenbogen SE, Dwyer MA, Ogba N, CGC, CGC, CGC (2019). NCCN guidelines insights: genetic/familial high-risk assessment: colorectal, version 2.2019. J Natl Compr Cancer Netw.

[9] Chapman-Davis E, Zhou ZN, Fields JC, Frey MK, Jordan B, Sapra KJ, Chatterjee-Paer S, Carlson AD, Holcomb KM (2021). Racial and ethnic disparities in genetic testing at a hereditary breast and ovarian cancer center. J Gen Intern Med.

[10] Hallenstein LG, Sorensen C, Hodgson L, Wen S, Westhuyzen J, Hansen C, Last ATJ, Amalaseelan JV, Salindera S, Ross W, Spigelman AD, Shakespeare TP, Aherne NJ (2021). Assessment of genetic referrals and outcomes for women with triple negative breast cancer in regional cancer centres in Australia. Hered Cancer Clin Pract..

[11] Peterson JM, Pepin A, Thomas R, Biagi T, Stark E, Sparks AD, Johnson K, Kaltman R (2020). Racial disparities in breast cancer hereditary risk assessment referrals. J Genet Couns.

[12] McKinney LP, Gerbi GB, Caplan LS, Claridy MD, Rivers BM (2020). Predictors of genetic beliefs toward cancer risk perceptions among adults in the United States: implications for prevention or early detection. J Genet Couns.

[13] Hoskovec JM, Bennett RL, Carey ME, DaVanzo JE, Dougherty M, Hahn SE (2018). Projecting the supply and demand for certified genetic counselors: a workforce study. J Genet Couns.

[14] Knerr S, Bowles EJA, Leppig KA, Buist DSM, Gao H, Wernli KJ (2019). Trends in BRCA test utilization in an integrated health system, 2005-2015. J Natl Cancer Inst.

[15] Aitken L, Warwick L, Davis A (2017). Breast and ovarian cancer referrals to the ACT genetic service: are we meeting guidelines?. Intern Med J.

[16] Gay EA, Byers PH, Bennett RL, Bird TD, Hisama FM (2019). Trends over 42 years in the adult medical genetics clinic at the University of Washington. Genet Med..

[17] Amendola LM, Berg JS, Horowitz CR, Angelo F, Bensen JT, Biesecker BB, Biesecker LG, Cooper GM, East K, Filipski K, Fullerton SM, Gelb BD, Goddard KAB, Hailu B, Hart R, Hassmiller-Lich K, Joseph G, Kenny EE, Koenig BA, Knight S, Kwok PY, Lewis KL, McGuire A, Norton ME, Ou J, Parsons DW, Powell BC, Risch N, Robinson M, Rini C, Scollon S, Slavotinek AM, Veenstra DL, Wasserstein MP, Wilfond BS, Hindorff LA, Plon SE, Jarvik GP, CSER consortium (2018). The clinical sequencing evidence-generating research consortium: integrating genomic sequencing in diverse and medically underserved populations. Am J Hum Genet.

[18] Hampel H, Bennett RL, Buchanan A, Pearlman R, Wiesner GL (2015). Guideline development group, et al. a practice guideline from the American College of Medical Genetics and Genomics and the National Society of genetic counselors: referral indications for cancer predisposition assessment. Genet Med..

[19] Harris PA, Taylor R, Minor BL, Elliott V, Fernandez M, O'Neal L (2019). The REDCap consortium: Building an international community of software platform partners. J Biomed Inform.

[20] Hunter JE, Arnold KA, Cook JE, Zepp J, Gilmore MJ, Rope AF, Davis JV, Bergen KM, Esterberg E, Muessig KR, Peterson SK, Syngal S, Acheson L, Wiesner G, Reiss J, Goddard KAB (2017). Universal screening for lynch syndrome among patients with colorectal cancer: patient perspectives on screening and sharing results with at-risk relatives. Familial Cancer.

[21] Evaluation of Genomic Applications in Practice Prevention Working Group (2009). Recommendations from the EGAPP working group: genetic testing strategies in newly diagnosed individuals with colorectal cancer aimed at reducing morbidity and mortality from lynch syndrome in relatives. Genet Med..

[22] Hampel H (2018). Population screening for hereditary colorectal Cancer. Surg Oncol Clin N Am.

[23] Wang A, McCracken J, Li Y, Xu L (2018). The practice of universal screening for lynch syndrome in newly diagnosed endometrial carcinoma. Health Sci Rep.

[24] Forbes C, Fayter D, de Kock S, Quek RG (2019). A systematic review of international guidelines and recommendations for the genetic screening, diagnosis, genetic counseling, and treatment of BRCA-mutated breast cancer. Cancer Manag Res.

[25] Hovick SR, Bevers TB, Vidrine JI, Kim S, Dailey PM, Jones LA, Peterson SK (2017). User perceptions and reactions to an online Cancer risk assessment tool: a process evaluation of Cancer risk check. J Cancer Educ.

[26] Li W, Murray MF, Giovanni MA (2019). Obtaining a genetic family history using computer-based tools. Curr Protoc Hum Genet.

[27] Ritchie JB, Bellcross C, Allen CG, Frey L, Morrison H, Schiffman JD, Welch BM (2021). Evaluation and comparison of hereditary cancer guidelines in the population. Hered Cancer Clin Pract.

[28] Moyer VA, U. S. Preventive Services Task Force (2014). Risk assessment, genetic counseling, and genetic testing for BRCA-related cancer in women: U.S. Preventive Services Task Force recommendation statement. Ann Intern Med.

[29] Lohn Z, Fok A, Richardson M, Derocher H, Mung SW, Nuk J, et al. Large-scale group genetic counseling: evaluation of a novel service delivery model in a Canadian hereditary cancer clinic. J Genet Couns. 2021. 10.1002/jgc4.1512. 10.1002/jgc4.1512 34596310

[30] Hann KEJ, Freeman M, Fraser L, Waller J, Sanderson SC, Rahman B (2017). Awareness, knowledge, perceptions, and attitudes towards genetic testing for cancer risk among ethnic minority groups: a systematic review. BMC Public Health.

[31] Muller C, Lee SM, Barge W, Siddique SM, Berera S, Wideroff G, Tondon R, Chang J, Peterson M, Stoll J, Katona BW, Sussman DA, Melson J, Kupfer SS (2018). Low referral rate for genetic testing in racially and ethnically diverse patients despite universal colorectal cancer screening. Clin Gastroenterol Hepatol.

[32] Boitano TKL, Barrington DA, Batra S, McGwin G, Turner TB, Farmer MB, Brown AM, Straughn MJ, Leath CA (2019). Differences in referral patterns based on race for women at high-risk for ovarian cancer in the southeast: results from a gynecologic Cancer risk assessment clinic. Gynecol Oncol.

[33] DeFrancesco MS, Waldman RN, Pearlstone MM, Karanik D, Bernhisel R, Logan J (2018). Hereditary cancer risk assessment and genetic testing in the community-practice setting. Obstet Gynecol.

[34] Kne A, Zierhut H, Baldinger S, Swenson KK, Mink P, Veach PM (2017). Why is cancer genetic counseling underutilized by women identified as at risk for hereditary breast cancer? Patient perceptions of barriers following a referral letter. J Genet Couns.

[35] Greenberg S, Buys SS, Edwards SL, Espinel W, Fraser A, Gammon A, Hafen B, Herget KA, Kohlmann W, Roundy C, Sweeney C (2019). Population prevalence of individuals meeting criteria for hereditary breast and ovarian cancer testing. Can Med.

[36] Hurtado-de-Mendoza A, Graves K, Gomez-Trillos S, Anderson L, Campos C, Evans C (2018). Provider's perceptions of barriers and facilitators for Latinas to participate in genetic cancer risk assessment for hereditary breast and ovarian cancer. Healthcare (Basel).

[37] Garcia C, Harrison K, Ring KL, Sullivan MW, Rauh LA, Modesitt SC (2019). Genetic counseling referral for ovarian cancer patients: a call to action. Familial Cancer.

[38] Rao SR, Breen N, Graubard BI (2016). Trends in black-white disparities in breast and colorectal cancer screening rates in a changing screening environment: the Peters-Belson approach uing United States national health interview surveys 2000-2010. Med Care.

[39] Institute of Medicine (US) Committee on Understanding and Eliminating Racial and Ethnic Disparities in Health Care. Unequal Treatment: Confronting Racial and Ethnic Disparities in Health Care (2003). In: appendix D. Racial disparities in health care: highlights from focus group findings [internet].

[40] United States Census Bureau (2016). Americans are visiting the doctor less frequently, Census Bureau reports 2012 [updated may 19].

[41] Silver MI, Klein W, Samimi G, Minasian L, Loud J, Roberts MC (2019). Concordance with BRCA1/2 testing guidelines among women in the health of women (HOW) study((R)). Breast Cancer Res Treat.

[42] Stuckey A, Febbraro T, Laprise J, Wilbur JS, Lopes V, Robison K (2016). Adherence patterns to national comprehensive cancer network guidelines for referral of women with breast cancer to genetics professionals. Am J Clin Oncol.

[43] Febbraro T, Robison K, Wilbur JS, Laprise J, Bregar A, Lopes V, Legare R, Stuckey A (2015). Adherence patterns to National Comprehensive Cancer Network (NCCN) guidelines for referral to cancer genetic professionals. Gynecol Oncol.

[44] Yedjou CG, Sims JN, Miele L, Noubissi F, Lowe L, Fonseca DD (2019). Health and racial disparity in breast cancer. Adv Exp Med Biol.

[45] Rolnick SJ, Rahm AK, Jackson JM, Nekhlyudov L, Goddard KA, Field T (2011). Barriers in identification and referral to genetic counseling for familial cancer risk: the perspective of genetic service providers. J Genet Couns.

[46] Laforest F, Kirkegaard P, Mann B, Edwards A (2019). Genetic cancer risk assessment in general practice: systematic review of tools available, clinician attitudes, and patient outcomes. Br J Gen Pract.

